# Impact of body mass index on in-hospital outcomes in patients receiving leadless pacemakers: A five-category analysis

**DOI:** 10.1016/j.hroo.2024.09.017

**Published:** 2024-10-01

**Authors:** Rajveer Sagoo, Navraj S. Sagoo, Ali S. Haider, Mohanakrishnan Sathyamoorthy

**Affiliations:** 1 College of Science and Engineering, Texas Christian University, Fort Worth, Texas; 2 Department of Internal Medicine, University of Tennessee Health Science Center, Chattanooga, Tennessee; 3 Rice University, Houston, Texas; 4 Department of Internal Medicine, Burnett School of Medicine at Texas Christian University (TCU) and Consultants in Cardiovascular Medicine and Science, Fort Worth, Texas

**Keywords:** Body mass index, Cardiovascular outcomes, In-hospital mortality, Leadless pacemaker, National Inpatient Sample (NIS), Obesity paradox

## Abstract

**Background:**

The adoption of leadless pacemakers (LPMs) is increasing, yet the impact of body mass index (BMI) on procedural outcomes remains underexplored.

**Objective:**

The purpose of this study was to explore the impact of BMI on in-hospital outcomes for patients receiving LPM implantation.

**Methods:**

Data from the National Inpatient Sample from 2018–2021 were analyzed for patients older than 18 years who underwent LPM implantation, with specific inclusion and exclusion criteria applied. Patients were identified using *International Classification of Diseases 10th Revision* codes and categorized into BMI groups: underweight, normal, overweight, obese, and morbidly obese. The primary outcome assessed was in-hospital mortality. Secondary outcomes included blood transfusion, pericardial complications, infection/inflammation, removal/revision, and other complications.

**Results:**

The study included 3832 patients who underwent LPM implantation between 2018 and 2021, weighted to represent 19,610 patients, with 3540 having an appropriate BMI designation. Mortality was lower in the obese group (2.3%) compared to the nonobese group (2.7%) (adjusted odds ratio [aOR] 0.462, 95% confidence interval [CI] 0.259–0.623, *P* = .009). Compared to the normal weight group, those categorized as overweight, obese, and morbidly obese demonstrated a lower risk of in-hospital mortality (aOR 0.432, 95% CI 0.299–0.734, *P* = .009; aOR 0.465, 95% CI 0.238–0.721, *P* <.001; aOR 0.299, 95% CI 0.153–0.586, *P* <.001, respectively).

**Conclusion:**

These findings support the existence of the obesity paradox in patients with LPM implantation, where higher BMI categories are associated with improved mortality outcomes, meeting our prespecified primary endpoint. Further studies are needed to clarify the mechanisms behind these observations.

**Key Findings**

▪ Overweight, obese, and morbidly obese patients who underwent leadless pacemaker implantation exhibited a lower risk of in-hospital mortality compared to normal weight patients.
▪ Obese and morbidly obese patients who underwent leadless pacemaker implantation had a lower risk of pericardial complications compared to normal weight patients.
▪ Morbidly obese patients who underwent leadless pacemaker implantation showed a lower risk of bleeding complications but a higher risk of device removal/revision compared to normal weight patients.

## Introduction

Leadless pacemakers (LPMs) represent a significant advancement in the field of cardiac rhythm management, offering numerous benefits over traditional transvenous pacemakers (TVPs).^1^, ^2^, ^3^, ^4^ Unlike TVPs, LPMs eliminate the need for leads, thereby reducing the risk of lead-related complications such as infections, lead dislodgment, and venous thrombosis.^5^^,^^6^ Additionally, the minimally invasive implantation procedure of LPMs, which involves delivery via a catheter through the femoral vein, is associated with reduced procedural complications and quicker recovery times,^7^ with iterative optimization in delivery catheter design enhancing maneuverability. Since their approval by the Food and Drug Administration in 2016, the utilization of LPMs has increased, particularly in patients who are at higher risk for complications associated with TVPs, such as those with recurrent infections, limited venous access, or high risk of lead dislodgment.^8^ Despite the growing use of LPMs, the impact of body mass index (BMI) on the outcomes of patients receiving these devices remains underexplored. BMI is a widely used metric to classify individuals based on body weight relative to height and is known to influence the prognosis of various cardiovascular conditions.^9^ However, its applicability to patients undergoing LPM implantation has not been investigated. This study aimed to assess whether BMI impacts outcomes in patients undergoing LPM implantation by comparing in-hospital outcomes across 5 BMI categories: underweight, normal weight, overweight, obese, and morbidly obese. By using the National Inpatient Sample (NIS) database from 2018 to 2021, which provides a nationally representative sample of hospitalized patients in the United States, we sought to provide a comprehensive analysis of how BMI influences the risks of mortality, ischemic cerebrovascular accident, cardiogenic shock, acute venous thromboembolism, cardiac complications, renal complications, bleeding complications, and the need for blood transfusions in patients who underwent LPM implantation. This analysis will advance knowledge on the association between BMI and outcomes in patients receiving LPMs, potentially informing clinical decision-making and patient selection strategies.

## Methods

This study used data from the NIS database, part of the Healthcare Cost and Utilization Project sponsored by the Agency for Healthcare Research and Quality. The NIS is the largest publicly available all-payer inpatient health care database in the United States, representing approximately 20% of inpatient discharges from U.S. hospitals. The database is designed to produce national estimates of inpatient care and includes a comprehensive set of discharge-level data such as patient demographics, diagnoses, procedures, discharge status, length of stay, and total charges. Each discharge record is weighted to allow for national estimates, ensuring the data are representative of the U.S. inpatient population. The NIS data are publicly available and de-identified; therefore, the need for informed consent and Institutional Review Board approval is waived. The NIS adheres to the 2013 Declaration of Helsinki for the conduct of human research.

Patients aged 18 years or older who underwent LPM implantation between 2018 and 2021 were identified using *International Classification of Diseases 10th Revision* (ICD-10) procedure code 02HK3NZ. The study focused on patients with complete data on BMI, categorized into 5 groups: underweight (≤19.9); normal weight (20–24.9); overweight (25–29.9); obese (30–34.9); and morbidly obese (≥35).To ensure a homogeneous study population and minimize potential confounding factors, several inclusion and exclusion criteria were applied and identified through relevant ICD-10 CM/PCS codes as listed in Supplemental Table S1. Inclusion criteria comprised adult patients aged 18 years or older who underwent LPM implantation. Exclusion criteria included patients with a history of a cardiovascular implantable electronic device (CIED), to avoid confounding outcomes related to previous device implantation. Additionally, hospitalizations involving concurrent major cardiac procedures such as valve surgery, atrial fibrillation ablation, and revascularization procedures were excluded to prevent the confounding impact of these interventions on outcomes. Hospitalizations with missing BMI data also were excluded to ensure accurate categorization and analysis.

The primary outcome of interest was in-hospital mortality. Secondary outcomes included removal/revision, mechanical complications, infection/inflammation, cardiogenic shock, venous thromboembolism, pericardial complications, renal complications, bleeding complications, and the need for blood transfusion. Pericardial complications included noninfective acute pericarditis, noninflammatory pericardial effusion, nontraumatic hemopericardium, cardiac tamponade, and unspecified pericardial complications. Renal complications included acute kidney injury. Bleeding complications covered periprocedural bleeding, postprocedural anemia, and hemoperitoneum/retroperitoneal bleeding. These complications were identified using relevant ICD-10 codes, which are listed in Supplemental Table S1. Descriptive statistics were used to summarize patient demographics and clinical characteristics across BMI categories.

For univariate comparisons of categorical variables, χ^2^ tests with Yates continuity correction or Fisher exact test were used. Continuous variables, such as age, are summarized as mean ± SD. The normality of age distribution was assessed using the Kolmogorov-Smirnov test, which indicated that the data did not follow a normal distribution. Consequently, the Mann-Whitney *U* test was used for comparing age between obese and nonobese groups.

To adjust for potential confounders, 2 multivariate logistic regression models were constructed. The first model compared all BMI categories against the outcomes of interest, adjusting for variables such as age, sex, comorbidities (including diabetes, hypertension, chronic kidney disease, chronic liver disease, cancer, smoking status, chronic pulmonary disease, and congestive heart failure). The second model dichotomized BMI into obese (BMI ≥30) and nonobese (BMI <30) groups and compared these groups against the same outcomes, using the same set of confounders. Adjusted odds ratios (aORs) with 95% confidence intervals (CIs) were calculated for each outcome in both models. All statistical analyses were conducted using SPSS Version 25 (SPSS Inc., Chicago, IL), and *P* <.05 was considered statistically significant. By using these rigorous statistical methods, the study aimed to robustly assess the relationship between BMI and in-hospital outcomes in patients undergoing LPM implantation.

## Results

**Figure 1:**
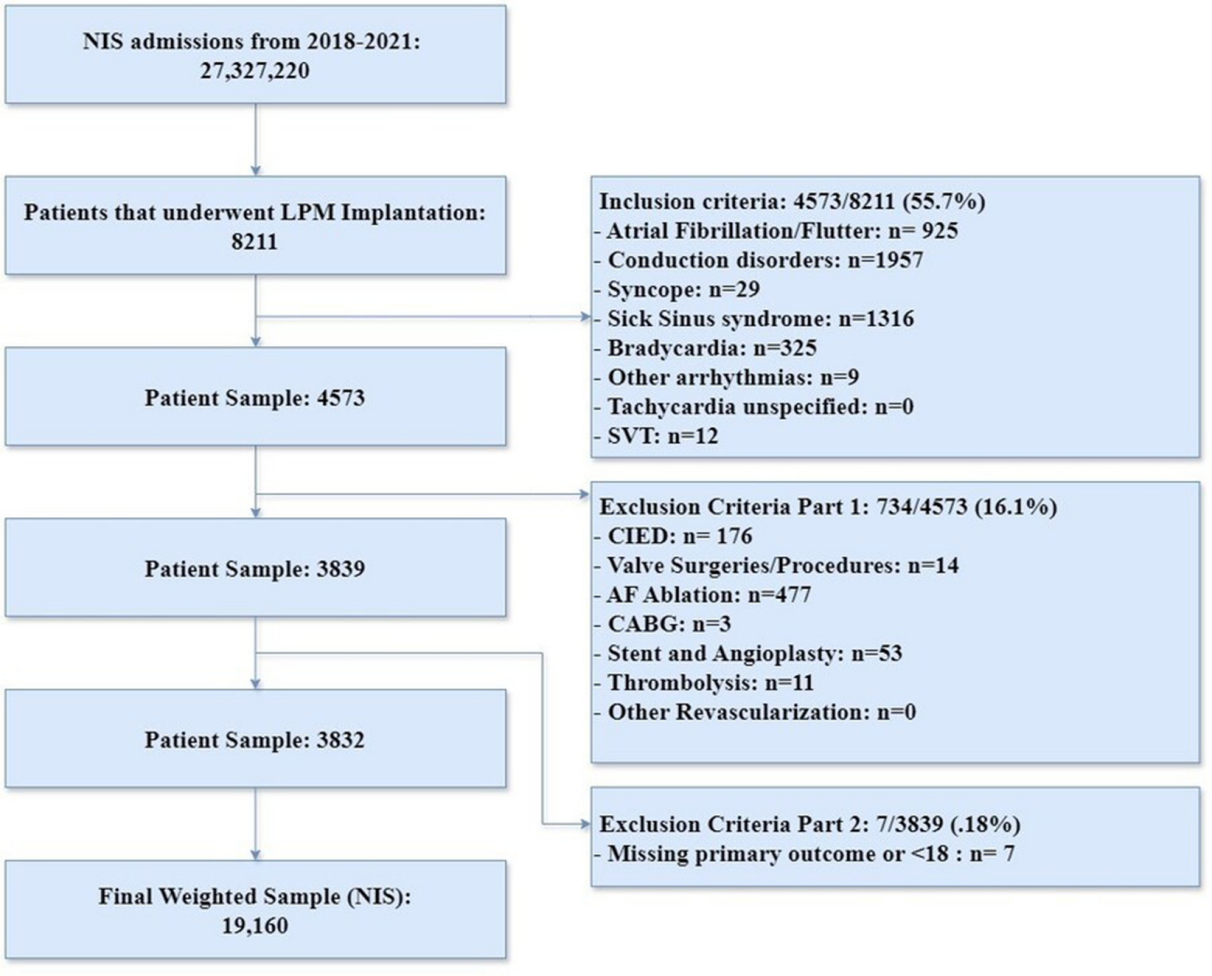
Diagram of patient sample selection detailing inclusion and exclusion criteria from initial National Inpatient Sample (NIS) admissions to a final weighted sample. AF = atrial fibrillation; CABG = coronary artery bypass graft; CIED = cardiovascular implantable electronic device; LPM = leadless pacemaker; SVT = supraventricular tachycardia.

Application of inclusion and exclusion criteria resulted in a sample size of 3832 patients who underwent LPM implantation between 2018 and 2021. Sample weighting (per NIS recommendation) resulted in 19,610 patients (Figure 1). From this cohort, 3540 patients had an appropriate BMI designation. Among these patients, 515 (15.9%) were classified as underweight, 305 (9.4%) as normal weight, 305 (9.4%) as overweight, 800 (24.7%) as obese, and 1615 (49.8%) as morbidly obese. Furthermore, significant differences in demographics and comorbid conditions were observed across the BMI categories (Table 1). Median age differed significantly across BMI categories. Underweight patients had the highest median [interquartile range] age of 84 [77–89] years, whereas morbidly obese patients had the lowest median age of 72 [63–73] years (*P* <.001). Female patients were more prevalent in the underweight and morbidly obese groups, representing 53.4% and 49.8% of these populations, respectively, compared to 37.7% in the overweight group (*P* <.001). The prevalence of diabetes, hyperlipidemia, and hypertension increased with higher BMI categories. Specifically, diabetes was present in 21.4% of underweight patients and 58.5% of morbidly obese patients (*P* <.001). Similarly, hypertension was present in 75.7% of underweight patients and 91.6% of morbidly obese patients (*P* <.001).

**Table 1:** Baseline demographics and comorbidities of study population

BMI, kg/m^2^						
Variable	Underweight (≤19.9) (n = 515)	Normal weight (20–24.9) (n = 305)	Overweight (25–29.9) (n = 305)	Obese (30–34.9) (n = 800)	Morbidly obese (≥35) (n = 1615)	*P* value
Age, y	84 [77–89]	82 [75–87]	81 [74–87]	76 [69–82]	72 [63–78]	<.001
Female	275 (53.4)	145 (48.3)	115 (37.7)	330 (41.3)	805 (49.8)	<.001
Diabetes	110 (21.4)	95 (31.1)	135 (44.3)	405 (50.6)	945 (58.5)	<.001
HLD	225 (43.7)	140 (45.9)	215 (70.5)	505 (63.1)	1005 (62.2)	<.001
Hypertension	390 (75.7)	225 (73.8)	275 (90.2)	710 (88.8)	1480 (91.6)	<.001
CHF	180 (35.0)	125 (41)	120 (39.3)	285 (35.6)	825 (51.1)	<.001
Chronic CAD	20 (3.9)	10 (3.3)	10 (3.3)	25 (3.1)	55 (3.4)	.839
Chronic ESRD	135 (26.2)	95 (31.1)	145 (47.5)	295 (36.9)	645 (39.9)	<.001
Cancer	55 (10.7)	35 (11.5)	35 (11.5)	80 (10.0)	80 (5.0)	<.001
Chronic pulmonary disease	110 (21.4)	60 (19.7)	50 (16.4)	185 (23.1)	450 (27.9)	<.001
Alcohol abuse	15 (2.9)	5 (1.6)	5 (1.6)	10 (1.3)	20 (1.2)	.144
Smoker	175 (34.0)	110 (36.1)	120 (39.3)	290 (36.3)	515 (31.9)	<.001
Chronic liver disease	0	20 (6.6)	5 (1.6)	5 (0.6)	45 (2.8)	<.001
White	400 (81.6)	245 (83.1)	225 (73.8)	630 (78.8)	1220 (78.5)	<.001
Black	40 (8.2)	15 (5.1)	50 (16.4)	80 (10.0)	175 (11.3)	
Hispanic	35 (7.1)	20 (6.8)	10 (3.3)	60 (7.5)	85 (5.5)	
Asian/Pacific Islander	10 (2.0)	10 (3.4)	10 (3.3)	10 (1.3)	20 (1.3)	
Native American	0	0	0	0	10 (0.1)	
Other	5 (1.0)	5 (1.7)	10 (3.3)	20 (2.5)	45 (2.9)	

Age is given as median [interquartile range]. All other values given as n (%) unless otherwise indicated.

BMI = body mass index; CAD = coronary artery disease; CHF = congestive heart failure; ESRD = end-stage renal disease; HLD = hyperlipidemia.

Table 2 lists the dichotomized logistic regression results comparing obese (BMI >30 kg/m^2^) and nonobese (BMI <30 kg/m^2^) groups. Mortality was lower in the obese group (2.3%) compared to the nonobese group (2.7%) (aOR 0.462, 95% CI 0.259–0.623, *P* = .009). Pericardial complications were also less frequent in the obese group (3.3%) compared to the nonobese group (3.6%) (aOR 1.488, 95% CI 1.246–1.634, *P* = .007). Bleeding complications were less common in the obese group (n = 110 [4.6%]) compared to the nonobese group (n = 65 [5.8%]) (aOR 0.834, 95% CI 0.634–0.972, *P* <.001). The incidence of cardiogenic shock was higher in the obese group (3.7%) compared to the nonobese group (3.1%) (aOR 1.435, 95% CI 1.232–1.634, *P* = .014). No significance was observed in venous thromboembolism (*P* = .009), blood transfusion (*P* = .130), renal complications (*P* = .418), and removal/revision (*P* = .102). Mechanical complications and infection/inflammation could not be reported because of NIS guidelines, which restrict reporting for samples for which n <11.

**Table 2:** Dichotomized logistic regression of leadless pacemaker outcomes: obese (BMI >30 kg/ m^2^) vs nonobese (BMI <30 kg/m^2^)

Outcomes	Nonobese (n = 1125)	Obese (n = 2415)	OR (95% CI)	P value	aOR (95% CI)	P value
Mortality	30 (2.7)	55 (2.3)	0.634 (0.421–0.851)	.012	.462 (0.259–0.623)	.009
Venous thromboembolism	30 (2.7)	50 (2.1)	0.753 (0.442–1.005)	.464	0.954 (0.756–1.124)	.503
Cardiogenic shock	25 (3.1)	90 (3.7)	1.643 (1.369–1.834)	.026	1.435 (1.232–1.634)	.014
Blood transfusion	25 (2.2)	80 (3.3)	0.904 (0.645–1.339)	.146	1.488 (1.246–1.754)	.130
Pericardial complications	40 (3.6)	80 (3.3)	0.734 (0.531–0.934)	.009	0.933 (0.874–1.021)	.007
Renal complications	270 (24.4)	585 (24.2)	1.522 (0.945–1.745)	.401	1.041 (0.853–1.271)	.418
Bleeding complications	65 (5.8)	110 (4.6)	0.557 (0.346–0.745)	<.001	0.834 (0.634–0.972)	<.001
Removal/revision	15 (1.3)	35 (1.4)	1.754 (1.356–1.935)	.115	1.294 (1.129–1.456)	.102
Mechanical complications	NR	NR	NR	NR	NR	NR
Infection/inflammation	NR	NR	NR	NR	NR	NR

Value are given as n (%) unless otherwise indicated.

aOR = adjusted odds ratio; BMI = body mass index; CI = confidence interval; NR = not reportable (n <11); OR = odds ratio.

Table 3 lists the occurrence and risk of various complications across different BMI categories compared to the normal BMI group (20–24.9 kg/m^2^). Higher risk of mortality was present within the underweight group (aOR 1.283, 95% CI 1.082–1.655, *P* = .005), and a lower risk was observed in the overweight (aOR 0.432, 95% CI 0.299–0.734, *P* = .009), obese (aOR 0.465, 95% CI 0.238–0.721, *P* <.001), and morbidly obese (aOR 0.299, 95% CI 0.153–0.586, *P* <.001) groups. Higher risk of pericardial complications was observed within the obese (aOR 2.655, 95% CI 2.352–2.841, *P* = .035) and morbidly obese (aOR 2.940, 95% CI 2.423–3.089, *P* = .036) groups. The risk of bleeding complications was lower in the obese (aOR 0.832, 95% CI 0.701–0.992, *P* = .045) and morbidly obese (aOR 0.845, 95% CI 0.681–1.002, *P* = .034) groups. The risk of removal/revision was higher in the morbidly obese group (aOR 1.289, 95% CI 1.089–1.453, *P* = .019).

**Table 3:** Leadless pacemaker complications stratified by BMI

BMI, kg/m^2^												
Outcomes	Underweight (>19) (n = 515)			Overweight (25–29.9) (n = 305)			Obese (30–34.9) (n = 800)			Morbidly obese (≥35) (n = 1615)		
	n (%)	OR (95% CI)	aOR (95% CI)	n (%)	OR (95% CI)	aOR (95% CI)	n (%)	OR (95% CI)	aOR (95% CI)	n (%)	OR (95% CI)	aOR (95% CI)
Mortality	20 (3.9)	1.412 (1.105–1.852)*P* =.014	1.283 (1.082–1.655)P = .005	NR	0.488 (0.328–0.901)P = .015	0.432 (0.299–0.734)*P* = .009	15 (1.9)	0.511 (0.261–0.803)P = .003	0.465 (0.238–0.721)*P* <.001	40 (2.5)	0.442 (0.178–0.615)P <.001	0.299 (0.153–0.586)*P* <.001
Venous thromboembolism	15 (2.9)	0.825 (0.492–1.486)*P* = .413	0.750 (0.425–1.231)P = .500	NR	0.685 (0.362–1.003)P = .621	0.728 (0.239–1.220)*P* = .577	20 (2.5)	1.065 (0.576–1.389)P = .922	1.032 (0.555–1.341)*P* = .941	35 (2.2)	1.015 (0.485–1.690)P = .891	0.940 (0.423–1.589)*P* = .879
Cardiogenic shock	15 (2.9)	0.953 (0.451–1.611)*P* = .665	0.871 (0.401–1.394)P = .728	NR	0.389 (0.148–0.774)P = .067	0.342 (0.116–0.533)*P* = .052	15 (1.9)	0.628 (0.290–0.920)P = .184	0.559 (0.253–.836)*P* = .151	75 (4.6)	1.620 (0.856–1.912)P = .215	1.561 (0.802–2.037)*P* = .190
Blood transfusion	20 (3.9)	2.080 (1.582–2.731)*P* = .203	2.231 (1.765–2.416)P = .119	NR	1.312 (0.871–1.328)P = .964	1.234 (0.798–1.547)*P* = .994	25 (3.1)	1.435 (0.902–2.221)P = .398	1.587 (0.988–2.084)*P* = .362	40 (2.5)	1.930 (1.350–2.270)P = .225	1.849 (1.307–2.134)*P* = .210
Pericardial complications	20 (3.9)	1.067 (0.822–1.492)*P* = .372	1.155 (.929–1.231)P = .500	15 (4.9)	2.119 (1.765–2.511)P = .199	2.242 (1.939–2.420)*P* = .154	30 (3.8)	2.470 (2.100–2.860)P = .061	2.655 (2.352–2.841)*P* = .035	35 (2.2)	3.105 (2.470–3.255)P = .041	2.940 (2.423–3.089)*P* = .036
Renal complications	125 (24.3)	1.157 (0.783–1.596)*P* = .253	1.283 (0.887–1.457)P = .186	85 (27.9)	1.503 (1.016–2.245)P = .138	1.402 (0.935–2.103)*P* = .102	160 (20)	0.998 (0.675–1.470)P = .896	1.038 (0.726–1.482)*P* = .840	425 (26.3)	1.628 (1.197–1.990)P = .341	1.581 (1.130–1.812)*P* = .322
Bleeding complications	35 (6.8)	0.718 (0.531–1.370)*P* = .305	0.802 (0.640–1.465)P = .474	NR	0.642 (0.249–1.085)P = .093	0.495 (0.223–0.998)*P* = .084	35 (4.4)	0.790 (0.620–1.005)P = .058	0.832 (0.701–0.992)*P* = .045	55 (3.4)	0.794 (0.661–1.025)P = .041	0.845 (0.681–0.997)*P* = .034
Removal/revision	NR	0.937 (0.711–1.621)*P* = .553	0.845 (0.645–1.345)P = .435	NR	0.869 (0.493–1.479)P = .631	0.802 (0.456–1.355)*P* = .703	20 (2.5)	1.280 (0.951–1.702)P = .777	1.343 (1.013–1.645)*P* = .724	25 (1.5)	1.337 (1.102–1.482)P = .024	1.289 (1.089–1.435)*P* = .019
Mechanical complications	NR	NR	NR	NR	NR	NR	NR	NR	NR	NR	NR	NR
Infection/inflammation	NR	NR	NR	NR	NR	NR	NR	NR	NR	NR	NR	NR

Normal weight BMI (20–24.9 kg/m^2^) is used as the reference category.

Abbreviations as in Table 2.

## Discussion

In the last decade, several studies have examined major cardiovascular outcome endpoints in LPM implantation; however, none have examined the correlation of such outcomes with BMI.^1^^,^^10^, ^11^, ^12^, ^13^, ^14^, ^15^, ^16^ To our knowledge, our study is the first to associate the impact of BMI on LPM outcomes within the United States and reveals 3 significant key findings: (1) overweight, obese, and morbidly obese patients who underwent LPM implantation exhibited a lower risk of in-hospital mortality compared to normal weight patients; (2) obese and morbidly obese patients who underwent LPM implantation had a lower risk of pericardial complications compared to normal weight patients; and (3) morbidly obese patients who underwent LPM implantation showed a lower risk of bleeding complications but a higher risk of device removal/revision compared to normal weight patients.

Advances in CIEDs, specifically pacemakers, have sparked new discussions on the influence of BMI and patient selection. A notable correlation has emerged in the literature named the “obesity paradox,” wherein higher BMI is associated with better outcomes in certain cardiovascular conditions.^17^, ^18^, ^19^, ^20^ For example, a study by Attanasio et al^21^ found that obese patients (BMI >30) demonstrated significantly lower major complications compared to nonobese patients (BMI <30) undergoing CIED implantation (11 [4.4%] vs 62 [8.7%], *P* <.05). Furthermore, a study by Almani et al^22^ analyzing data from 2016–2018 demonstrated that obese patients who underwent TVP insertion exhibited lower inpatient mortality compared to nonobese patients (7.0% vs 10.8%). Consistent with this paradoxical association, our study found that patients classified as overweight, obese, and morbidly obese who underwent LPM implantation exhibited a lower risk of in-hospital mortality compared to normal weight patients. Additionally, in a dichotomized cohort, we found obese patients demonstrated lower in-hospital mortality compared to nonobese patients (30 [2.7%] vs 55 [2.3%], *P* = .009). In a pivotal prospective study using the Micra postapproval registry, several advantages of LPMs over TVPs implantation were examined.^12^ Over a period of 3 years, the rate of major complications observed with LPMs was 53% lower compared to traditional transvenous pacemakers, primarily due to the reduction of lead dislodgments and the need for system revisions. At 36-month follow-up, the system revision rate was significantly lower with Micra vs transvenous systems (3.2% vs 6.6%, *P* <.001) Furthermore, reduced infection rates due to the elimination of the subcutaneous pocket required for TVPs resulted in no Micra removals due to infection over the 5-year period. The minimally invasive nature of the LPM procedure offers clear benefits in procedural complications, especially for obese patients. Obese individuals often face higher risks with implantation because of the increased challenge of dissection complexity to create a suitable implant pocket.^23^ In contrast, we found that underweight patients demonstrated a higher risk of in-hospital mortality compared to normal weight patients. This may be attributed to their generally weaker overall health and potential comorbidities, such as malnutrition or frailty, which could exacerbate the stress of the procedure.

Our first key finding—that overweight, obese, and morbidly obese groups exhibited a lower risk of mortality—can be attributed to several factors. Increased adiposity correlates with a thicker anterior fat pad, which may potentially reduce pericardial complications.^24^^,^^25^ This protective layer may further mitigate mechanical stress and inflammation during the implantation procedure, contributing to better outcomes.^25^

Our second key finding that obese and morbidly obese patients had a lower risk of pericardial complications compared to their normal weight counterparts lends initial support to this assertion. Importantly, we classified pericardial complications as a composite of various related issues, detailed in the Supplemental Material. Although limited studies are available, our results align with recent research examining the correlation between obesity and specific pericardial complications. For example, a study conducted by Khan et al^26^ found that obesity was associated with a decreased risk of pericardial effusion requiring intervention after LPM implantation. Furthermore, Piccini et al^27^ reported that increasing BMI on a continuous basis and a history of atrial fibrillation were associated with a lower occurrence of pericardial effusion. As mentioned earlier, adiposity (through effects on the thickness and composition of the anterior fat pad) may confer an advantage in absorbing mechanical stresses during device implantation. Additionally, the altered immune response in obese individuals may modulate the acute inflammatory response typically involved in pericardial effusion and pericarditis.^28^ Importantly, awareness of potential pericardial complications, even in the absence of perforation, can prevent prolonged hospitalization and serious complications such as tamponade and constrictive pericarditis.

Our third key finding was that morbidly obese patients showed a lower risk of bleeding complications but a higher risk of device removal/revision. Bleeding complications were defined as a composite of periprocedural bleeding, postprocedural anemia, and hemoperitoneum/retroperitoneal bleeding. In accordance with our results, Attanasio et al^21^ found that both major and minor bleeding complications were lower in an obese (BMI >30) vs nonobese cohort (BMI <30) of patients who underwent cardiac device implantation. This protective effect may be attributed to enhanced platelet function and coagulation profiles in obese individuals.^29^ However, the increased risk of device removal or revision in morbidly obese patients undergoing LPM is a multifaceted challenge. The technical challenges of implanting the LPM in obese patients, such as difficulties in accessing vascular sites and securing the device, may contribute to a higher likelihood of complications. Furthermore, a study by Tseng et al^30^ found that lead dislodgment is more common in patients with certain risk factors, including obesity, which can increase the need for device revision. Although this study focused on traditional pacemakers and implantable cardioverter-defibrillators, the principles of device instability and mechanical complications are relevant to LPMs.^30^ A major advance in the latest versions of LPMs feature delivery catheters with more facile steerability, benching, and device deliverability that should help reduce complications at the time of implant.

### Study limitations

Notably, there are limitations inherent to the NIS database. (1) The reliance on ICD codes for disease and procedural identification may introduce potential inaccuracies, despite the Agency for Healthcare Research and Quality’s rigorous data quality control measures aimed at minimizing miscoding and ensuring data integrity. (2) The database encompasses only inpatient admissions, omitting outpatient encounters, and lacks longitudinal follow-up, which restricts our understanding of long-term outcomes and the dynamics of readmissions. (3) The NIS does not differentiate between different LPM platforms such as Micra and Aveir, which limits our ability to compare outcomes across these devices. (4) The NIS does not capture critical details such as medications and specific procedural data, including steps involved in the LPM implantation process such as operator experience, use of intraprocedural imaging, and contrast utilization. (5) The absence of detailed patient-level data, such as lifestyle factors, precludes more comprehensive risk stratification and understanding of the interactions between BMI and pacemaker outcomes.

## Conclusion

Our study highlights 3 key findings on the impact of BMI on LPM outcomes in the United States. (1) Overweight, obese, and morbidly obese patients had lower in-hospital mortality compared to normal weight patients. (2) Obese and morbidly obese patients had fewer pericardial complications compared to normal weight patients. (3) Morbidly obese patients had a lower risk of bleeding complications but a higher risk of device removal or revision compared to normal weight patients. These findings suggest that patients with higher BMI are likely to considerably benefit from LPM implantation in comparison to traditional lead-based devices. Future prospective studies and registry-based research should investigate the underlying mechanisms behind these outcomes to refine patient selection for LPMs and enhance long-term patient care.
