# Supplementary material for: Impact of body mass index on in-hospital outcomes in patients receiving leadless pacemakers: A five-category analysis
Source: Heart Rhythm O2. 2024 Oct 1;5(12):883–9. doi: 10.1016/j.hroo.2024.09.017 (PMC11721722; doi:10.1016/j.hroo.2024.09.017)
Supplement: Supplementary Material [file mmc1.docx]

**Supplementary Table**

**S1**

Sampling was based on the International Classification of Diseases, 10th Revision, Clinical Modification/Procedure Coding System (ICD-10 CM/PCS) codes.

**Initial Sample**

| **Variable** | **ICD-10** | **Code(s)** |
| --- | --- | --- |
| Leadless pacemaker implantation | PCS | 02HK3NZ |

**Inclusion Criteria (Principal Discharge Diagnosis)**

| **Variable** | **ICD-10** | **Code(s)** |
| --- | --- | --- |
| Atrial fibrillation and flutter | CM | I480, I481, I4811, I4819, I482, I4820, I4821, I4891, I483, I484, I4892 |
| Conduction disorders | CM | I440, I441, I442, I4430, I4439, I444, I445, I4460, I4469, I447, I450, I4510, I4519, I452, I453, I454, I455, I4589, I459 |
| Syncope | CM | R55, G9001 |
| Sick sinus syndrome | CM | I495 |
| Bradycardia | CM | R001 |
| Other arrhythmias | CM | I498, I499 |
| Tachycardia, unspecified | CM | R000 |
| SVT | CM | I471 |

**Exclusion Criteria**

| **Variable** | | **ICD-10** | **Code(s)** |
| --- | --- | --- | --- |
| H/o CIED | | CM | Z950, Z95810 |
| Valve surgeries/procedures | | PCS | 024F0xJ (7, 8, J, K), 024G0xJ (7, 8, J, K), 024J0xJ (7, 8, J, K), 025FxZZ (0, 3, 4), 025GxZZ (0, 3, 4), 025HxZZ (0, 3, 4), 025JxZZ (0, 3, 4),  027F0xZ (4, D, Z),  027F3xZ (4, D, Z),  027F4xZ (4, D, Z), 027G0xZ (4, D, Z),  027G3xZ (4, D, Z),  027G4xZ (4, D, Z), 027H0xZ (4, D, Z),  027H3xZ (4, D, Z),  027H4xZ (4, D, Z), 027J0xZ (4, D, Z),  027J3xZ (4, D, Z),  027J4xZ (4, D, Z), 02BF0Zx (X, Z), 02BF3Zx (X, Z), 02BF4Zx (X, Z), 02BG0Zx (X, Z), 02BG3Zx (X, Z), 02BG4Zx (X, Z), 02BH0Zx (X, Z), 02BH3Zx (X, Z), 02BH4Zx (X, Z), 02BJ0Zx (X, Z), 02BJ3Zx (X, Z), 02BJ4Zx (X, Z), 02CFxZZ (0, 3, 4), 02CGxZZ (0, 3, 4), 02CHxZZ (0, 3, 4), 02CJxZZ (0, 3, 4), 02NFxZZ (0, 3, 4), 02NGxZZ (0, 3, 4), 02NHxZZ (0, 3, 4), 02NJxZZ (0, 3, 4), 02QF0Zx (J, Z), 02QF3Zx (J, Z), 02QF4Zx (J, Z), 02QG0Zx (J, Z), 02QG3Zx (J, Z), 02QG4Zx (J, Z), 02QH0Zx (J, Z), 02QH3Zx (J, Z), 02QH4Zx (J, Z), 02QJ0Zx (J, Z), 02QJ3Zx (J, Z), 02QJ4Zx (J, Z), 02RF0xZ (7, 8, J, K), 02RF3xx (7H, 7Z, 8H, 8Z, JH, JZ, KH, KZ), 02RF4xZ (7, 8, J, K), 02RG0xZ (7, 8, J, K), 02RG3xx (7H, 7Z, 8H, 8Z, JH, JZ, KH, KZ), 02RG4xZ (7, 8, J, K), 02RH0xZ (7, 8, J, K), 02RH3xx (7H, 7Z, 8H, 8Z, JH, JZ, KH, KZ), 02RH4xZ (7, 8, J, K), 02RJ0xZ (7, 8, J, K), 02RJ3xx (7H, 7Z, 8H, 8Z, JH, JZ, KH, KZ), 02RJ4xZ (7, 8, J, K), 02UF0xx (7J, 7Z, 8J, 8Z, JJ, JZ, KJ, KZ), 02UF3xx (7J, 7Z, 8J, 8Z, JJ, JZ, KJ, KZ), 02UF4xx (7J, 7Z, 8J, 8Z, JJ, JZ, KJ, KZ), 02UG0xx (7J, 7Z, 8J, 8Z, JJ, JZ, KJ, KZ), 02UG3xx (7J, 7Z, 8J, 8Z, JJ, JZ, KJ, KZ), 02UG4xx (7J, 7Z, 8J, 8Z, JJ, JZ, KJ, KZ), 02UH0xx (7J, 7Z, 8J, 8Z, JJ, JZ, KJ, KZ), 02UH3xx (7J, 7Z, 8J, 8Z, JJ, JZ, KJ, KZ), 02UH4xx (7J, 7Z, 8J, 8Z, JJ, JZ, KJ, KZ), 02UJ0xx (7J, 7Z, 8J, 8Z, JJ, JZ, KJ, KZ), 02UJ3xx (7J, 7Z, 8J, 8Z, JJ, JZ, KJ, KZ), 02UJ4xx (7J, 7Z, 8J, 8Z, JJ, JZ, KJ, KZ) |
| AF Ablation | | PCS | 02563ZZ, 02573ZZ, 02583ZZ, 025S3ZZ, 025T3ZZ, 02560ZZ, 02564ZZ, 02570ZZ, 02574ZZ, 025S4ZZ, 025T4ZZ |
| Revascularization | Stent & Angioplasty | PCS | Percutaneous coronary stenting:  “Dilation” ICD wording:  027034x(6,Z), 027035x(6,Z), 027036x(6,Z), 027037x(6,Z), 02703Dx(6,Z), 02703Ex(6,Z), 02703Fx(6,Z), 02703Gx(6,Z), 027134x(6,Z), 027135x(6,Z), 027136x(6,Z), 027137x(6,Z), 02713Dx(6,Z), 02713Ex(6,Z), 02713Fx(6,Z), 02713Gx(6,Z), 027234x(6,Z), 027235x(6,Z), 027236x(6,Z), 027237x(6,Z), 02723Dx(6,Z), 02723Ex(6,Z), 02723Fx(6,Z), 02723Gx(6,Z), 027334x(6,Z), 027335x(6,Z), 027336x(6,Z), 027337x(6,Z), 02733Dx(6,Z), 02733Ex(6,Z), 02733Fx(6,Z), 02733Gx(6,Z)  “Insertion” ICD wording (only intraluminal device; excluded insertion of “other” device):  02H03DZ, 02H13DZ, 02H23DZ, 02H33DZ  Angioplasty:  02703Zx(6,Z), 02713Zx(6,Z), 02723Zx(6,Z), 02733Zx(6,Z) |
|  | Thrombolysis w/ cath | PCS | 3E07316 + 3E07317 |
|  | Thrombolysis peripheral | 99.10 | 3E03016, 3E03017, 3E03316, 3E03317 |
|  | CABG | PCS | 021008x(3,8,9,C,F,W),  021009x(3,8,9,C,F,W), 02100Ax(3,8,9,C,F,W), 02100Jx(3,8,9,C,F,W), 02100Kx(3,8,9,C,F,W), 02100Zx(3,8,9,C,F), 02103x(44,D4), 0210444, 021048x(3,8,9,C,F,W), 021049x(3,8,9,C,F,W), 02104Ax(3,8,9,C,F,W), 02104Jx(3,8,9,C,F,W), 02104Kx(3,8,9,C,F,W), 02104Zx(3,8,9,C,F), 02104D4, 021108x(3,8,9,C,F,W),  021109x(3,8,9,C,F,W), 02110Ax(3,8,9,C,F,W), 02110Jx(3,8,9,C,F,W), 02110Kx(3,8,9,C,F,W), 02110Zx(3,8,9,C,F), 02113x(44,D4), 0210444, 021148x(3,8,9,C,F,W), 021149x(3,8,9,C,F,W), 02114Ax(3,8,9,C,F,W), 02114Jx(3,8,9,C,F,W), 02114Kx(3,8,9,C,F,W), 02114Zx(3,8,9,C,F), 02114D4, 021208x(3,8,9,C,F,W),  021209x(3,8,9,C,F,W), 02120Ax(3,8,9,C,F,W), 02120Jx(3,8,9,C,F,W), 02120Kx(3,8,9,C,F,W), 02120Zx(3,8,9,C,F), 02123x(44,D4), 0212444, 021248x(3,8,9,C,F,W), 021249x(3,8,9,C,F,W), 02124Ax(3,8,9,C,F,W), 02124Jx(3,8,9,C,F,W), 02124Kx(3,8,9,C,F,W), 02124Zx(3,8,9,C,F), 02124D4, 021308x(3,8,9,C,F,W),  021309x(3,8,9,C,F,W), 02130Ax(3,8,9,C,F,W), 02130Jx(3,8,9,C,F,W), 02130Kx(3,8,9,C,F,W), 02130Zx(3,8,9,C,F), 02133x(44,D4), 0213444, 021348x(3,8,9,C,F,W), 021349x(3,8,9,C,F,W), 02134Ax(3,8,9,C,F,W), 02134Jx(3,8,9,C,F,W), 02134Kx(3,8,9,C,F,W), 02134Zx(3,8,9,C,F), 02134D4 |
|  | Other revascularizations | PCS | 027004x(6,Z), 027005x(6,Z), 027006x(6,Z), 027007x(6,Z), 02700Dx(6,Z), 02700Ex(6,Z), 02700Fx(6,Z), 02700Gx(6,Z), 02700Tx(6,Z),  02703Tx(6,Z), 027044x(6,Z), 027045x(6,Z), 027046x(6,Z), 027047x(6,Z), 02704Dx(6,Z), 02704Ex(6,Z), 02704Fx(6,Z), 02704Gx(6,Z), 02704Tx(6,Z), 027104x(6,Z), 027105x(6,Z), 027106x(6,Z), 027107x(6,Z), 02710Dx(6,Z), 02710Ex(6,Z), 02710Fx(6,Z), 02710Gx(6,Z), 02710Tx(6,Z),  02713Tx(6,Z), 027144x(6,Z), 027145x(6,Z), 027146x(6,Z), 027147x(6,Z), 02714Dx(6,Z), 02714Ex(6,Z), 02714Fx(6,Z), 02714Gx(6,Z), 02714Tx(6,Z), 027204x(6,Z), 027205x(6,Z), 027206x(6,Z), 027207x(6,Z), 02720Dx(6,Z), 02720Ex(6,Z), 02720Fx(6,Z), 02720Gx(6,Z), 02720Tx(6,Z),  02723Tx(6,Z), 027244x(6,Z), 027245x(6,Z), 027246x(6,Z), 027247x(6,Z), 02724Dx(6,Z), 02724Ex(6,Z), 02724Fx(6,Z), 02724Gx(6,Z), 02724Tx(6,Z), 027304x(6,Z), 027305x(6,Z), 027306x(6,Z), 027307x(6,Z), 02730Dx(6,Z), 02730Ex(6,Z), 02730Fx(6,Z), 02730Gx(6,Z), 02730Tx(6,Z), 02733Tx(6,Z), 027344x(6,Z), 027345x(6,Z), 027346x(6,Z), 027347x(6,Z), 02734Dx(6,Z), 02734Ex(6,Z), 02734Fx(6,Z), 02734Gx(6,Z), 02734Tx(6,Z), 02H00DZ, 02H04DZ, 02H10DZ, 02H14DZ, 02H20DZ, 02H24DZ, 02H30DZ, 02H34DZ, 02700Zx(6,Z), 02704Zx(6,Z), 02710Zx(6,Z), 02714Zx(6,Z), 02720Zx(6,Z), 02724Zx(6,Z), 02730Zx(6,Z), 02734Zx(6,Z) |

**BMI Categories and Comorbidities**

| **Variable** | **ICD-10** | **Code(s)** |
| --- | --- | --- |
| Underweight (BMI ≤ 19.9) | CM | Z681 |
| Normal weight (BMI 20-24.9) | CM | Z6820, Z6821, Z6822, Z6823, Z6824 |
| Overweight (BMI 25-29.9) | CM | Z6825, Z6826, Z6827, Z6828, Z6829 |
| Obese (BMI 30-34.9) | CM | Z6830, Z6831, Z6832, Z6833, Z6834 |
| Morbidly obese (BMI ≥ 35) | CM | Z6835, Z6836, Z6837, Z6838, Z6839, Z684 |
| Diabetes | CM | E08, E09, E10, E11, E12, E13, E0800, E0801, E0810, E0811, E0821, E0822, E0829, E08311, E08319, E08321, E083211, E083212, E083213, E083219, E08329, E083291, E083292, E083293, E083299, E08331, E083311, E083312, E083313, E083319, E08339, E083391, E083392, E083393, E083399, E08341, E083411, E083412, E083413, E083419, E08349, E083491, E083492, E083493, E083499, E08351, E083511, E083512, E083513, E083519, E083521, E083522, E083523, E083529, E083531, E083532, E083533, E083539, E083541, E083542, E083543, E083549, E083551, E083552, E083553, E083559, E08359, E083591, E083592, E083593, E083599, E0836, E0837X1, E0837X2, E0837X3, E0837X9, E0839, E0840, E0841, E0842, E0843, E0844, E0849, E0851, E0852, E0859, E08610, E08618, E08620, E08621, E08622, E08628, E08630, E08638, E08641, E08649, E0865, E0869, E088, E089, E0900, E0901, E0910, E0911, E0921, E0922, E0929, E09311, E09319, E09321, E093211, E093212, E093213, E093219, E09329, E093291, E093292, E093293, E093299, E09331, E093311, E093312, E093313, E093319, E09339, E093391, E093392, E093393, E093399, E09341, E093411, E093412, E093413, E093419, E09349, E093491, E093492, E093493, E093499, E09351, E093511, E093512, E093513, E093519, E093521, E093522, E093523, E093529, E093531, E093532, E093533, E093539, E093541, E093542, E093543, E093549, E093551, E093552, E093553, E093559, E09359, E093591, E093592, E093593, E093599, E0936, E0937X1, E0937X2, E0937X3, E0937X9, E0939, E0940, E0941, E0942, E0943, E0944, E0949, E0951, E0952, E0959, E09610, E09618, E09620, E09621, E09622, E09628, E09630, E09638, E09641, E09649, E0965, E0969, E098, E099, E1010, E1011, E1021, E1022, E1029, E10311, E10319, E10321, E103211, E103212, E103213, E103219, E10329, E103291, E103292, E103293, E103299, E10331, E103311, E103312, E103313, E103319, E10339, E103391, E103392, E103393, E103399, E10341, E103411, E103412, E103413, E103419, E10349, E103491, E103492, E103493, E103499, E10351, E103511, E103512, E103513, E103519, E103521, E103522, E103523, E103529, E103531, E103532, E103533, E103539, E103541, E103542, E103543, E103549, E103551, E103552, E103553, E103559, E10359, E103591, E103592, E103593, E103599, E1036, E1037X1, E1037X2, E1037X3, E1037X9, E1039, E1040, E1041, E1042, E1043, E1044, E1049, E1051, E1052, E1059, E10610, E10618, E10620, E10621, E10622, E10628, E10630, E10638, E10641, E10649, E1065, E1069, E108, E109, E1100, E1101, E1110, E1111, E1121, E1122, E1129, E11311, E11319, E11321, E113211, E113212, E113213, E113219, E11329, E113291, E113292, E113293, E113299, E11331, E113311, E113312, E113313, E113319, E11339, E113391, E113392, E113393, E113399, E11341, E113411, E113412, E113413, E113419, E11349, E113491, E113492, E113493, E113499, E11351, E113511, E113512, E113513, E113519, E113521, E113522, E113523, E113529, E113531, E113532, E113533, E113539, E113541, E113542, E113543, E113549, E113551, E113552, E113553, E113559, E11359, E113591, E113592, E113593, E113599, E1136, E1137X1, E1137X2, E1137X3, E1137X9, E1139, E1140, E1141, E1142, E1143, E1144, E1149, E1151, E1152, E1159, E11610, E11618, E11620, E11621, E11622, E11628, E11630, E11638, E11641, E11649, E1165, E1169, E118, E119, E1300, E1301, E1310, E1311, E1321, E1322, E1329, E13311, E13319, E13321, E133211, E133212, E133213, E133219, E13329, E133291, E133292, E133293, E133299, E13331, E133311, E133312, E133313, E133319, E13339, E133391, E133392, E133393, E133399, E13341, E133411, E133412, E133413, E133419, E13349, E133491, E133492, E133493, E133499, E13351, E133511, E133512, E133513, E133519, E133521, E133522, E133523, E133529, E133531, E133532, E133533, E133539, E133541, E133542, E133543, E133549, E133551, E133552, E133553, E133559, E13359, E133591, E133592, E133593, E133599, E1336, E1337X1, E1337X2, E1337X3, E1337X9, E1339, E1340, E1341, E1342, E1343, E1344, E1349, E1351, E1352, E1359, E13610, E13618, E13620, E13621, E13622, E13628, E13630, E13638, E13641, E13649, E1365, E1369, E138, E139 |
| Chronic pulmonary diseases | CM | J44, J45, J46, J410, J411, J418, J42, J430, J431, J432, J438, J439, J440, J441, J449 |
| HLD | CM | E78, E780, E7800, E7801, E781, E782, E783, E784, E7841, E7849, E785 |
| Cancer | CM | C00-C97 |
| Smoker | CM | F17, Z720, Z716, Z8643, Z87891, O9933, T652 |
| Chronic Coronary Artery Disease | CM | I255, I256, I25700, I25701, I25708, I25709, I25710, I25711, I25718, I25719, I25720, I25721, I25728, I25729, I25730, I25731, I25738, I25739, I25750, I25751, I25758, I25759, I25760, I25761, I25768, I25769, I25790, I25791, I25798, I25799, I25810, I25811, I25812, I2582, I2583, I2584, I2589, I259 |
| Hypertension | CM | I10, I110, I119, I120, I129, I130, I1310, I1311, I132, I150, I151, I152, I158, I159, I160, I161, I169 |
| Chronic/End-Stage Renal Disease | CM | N181, N182, N183, N1830, N1831, N1832, N184, N185, N186, N189 |
| Alcohol Abuse | CM | F1010, F1011, F10120, F10121, F10129, F10130, F10131, F10132, F10139, F1014, F10150, F10151, F10159, F10180, F10181, F10182, F10188, F1019, F1020, F1021, F10220, F10221, F10229, F10230, F10231, F10232, F10239, F1024, F10250, F10251, F10259, F1026, F1027, F10280, F10281, F10282, F10288, F1029, F10920, F10921, F10929, F10930, F10931, F10932, F10939, F1094, F10950, F10951, F10959, F1096, F1097, F10980, F10981, F10982, F10988, F1099 |
| Chronic Liver Disease (CLD) | CM | K700, K7010, K7011, K702, K7030, K7031, K7040, K7041, K709, K710, K7110, K7111, K712, K713, K714, K7150, K7151, K716, K717, K718, K719, K7210, K7211, K730, K731, K732, K738, K739, K740, K7400, K7401, K7402, K741, K742, K743, K744, K745, K7460, K7469, K7581 |

**Outcomes**

| **Variable** | **ICD-10** | **Code(s)** |
| --- | --- | --- |
| Cardiogenic shock | CM | R570 |
| Transfusion | PCS | 30230H, 30233H, 30233K, 30230K, 30233L, 30230L, 30233M, 30230M, 30233N, 30230N, 30233P, 30230P, 30233R, 30230R, 30233T, 30230T, 30233V, 30230V, 30233W, 30230W |
| Pericardial complications | CM | Non-infective acute pericarditis (I300, I308, I309)  Non-inflammatory Pericardial effusion (I313)  Non-traumatic hemopericardium (I312)  Cardiac tamponade (I314)  Unspecified pericardial complication (I319) |
| **Venous Thromboembolism** | | |
| Deep venous thrombosis | CM | Lower extremity DVT  I82401, I82402, I82403, I82409, I82411, I82412, I82413, I82419, I82421, I82422, I82423, I82429, I82431, I82432, I82433, I82439, I82441, I82442, I82443, I82449, I82451, I82452, I82453, I82459, I82461, I82462, I82463, I82469, I82491, I82492, I82493, I82499, I824Y1, I824Y2, I824Y3, I824Y9, I824Z1, I824Z2, I824Z3, I824Z9 |
| Pulmonary embolism | CM | I2601, I2602, I2609, I2690, I2692, I2693, I2694, I2699 |
| **Bleeding complications** |  |  |
| Periprocedural bleeding | CM | I97410, I97418, I9742, I97610, I97618, I97620, I97621, I9631, I97.638 |
| Post-procedural anemia | CM | D62 |
| Hemoperitoneum/Retroperitoneal bleeding | CM | K661 |
| **Renal complications** | | |
| Acute kidney injury | CM | N170, N171, N172, N178, N179 |
| **Device Complications** | | |
| Mechanical Complications (Breakdown and Displacement) | CM | T8211, T82111, T82111A, T82111D, T82111S, T82118, T82118A, T82118D, T82118S, T82119, T82119A, T82119D, T82119S, T82128, T82128A, T82128D, T82128S, T82129, T82129A, T82129D, T82129S, T8219, T82198, T82198A, T82198D, T82198S, T82199, T82199A, T82199D, T82199S |
| Removal/Revision | PCS | 02PA3NZ, 02WA3NZ. |
| Infection/inflammation | CM | T827, T827XA, T827XD, T827XS |
